# Chronic calcitriol supplementation improves the inflammatory profiles of circulating monocytes and the associated intestinal/adipose tissue alteration in a diet-induced steatohepatitis rat model

**DOI:** 10.1371/journal.pone.0194867

**Published:** 2018-04-23

**Authors:** Yen-Bo Su, Tzu-Hao Li, Chia-Chang Huang, Hung-Cheng Tsai, Shiang-Fen Huang, Yun-Cheng Hsieh, Ying-Ying Yang, Yi-Hsiang Huang, Ming-Chih Hou, Han-Chieh Lin

**Affiliations:** 1 Department of Medicine, Taipei Veterans General Hospital, Taipei, Taiwan; 2 Department of Medicine, National Yang-Ming University School of Medicine, Taipei, Taiwan; 3 Division of Allergy and Immunology, Taipei Veterans General Hospital, Taipei, Taiwan; 4 Institute of Clinical Medicine, National Yang-Ming University School of Medicine, Taipei, Taiwan; 5 Chia-Yi Branch of Taichung Veterans General Hospital, Chiayi, Taiwan; 6 Division of Infection, Taipei Veterans General Hospital, Taipei, Taiwan; 7 Division of Gastroenterology and Hepatology, Taipei Veterans General Hospital, Taipei, Taiwan; 8 Division of General Medicine, Taipei Veterans General Hospital, Taipei, Taiwan; IDIBAPS Biomedical Research Institute, SPAIN

## Abstract

Vitamin D deficiency and up-regulated TNFα-related signals are reported to be involved in abnormalities including intestinal hyper-permeability, bacterial translocation, systemic/portal endotoxemia, intestinal/adipose tissue/hepatic inflammation, and hepatic steatosis in nonalcoholic steatohepatitis (NASH). This study aims to explore the molecular mechanisms and effects of chronic calcitriol [1,25-(OH)_2_D_3_, hormonal form of vitamin D] on gut-adipose tissue-liver axis abnormalities using a high-fat diet (HFD)-fed rat model of NASH. In HFD-fed obese rats on a 10-week calcitriol (0.3 μg/kg/TIW) or vehicle treatment (NASH-vit. D and NASH-V rats) reigme, various *in vivo* and *in vitro* experiments were undertaken. Through anti-TNFα-TNFR1-NFκB signaling effects, chronic calcitriol treatment significantly restored plasma calcitriol levels and significantly improved vitamin D receptor (VDR) expression in monocytes and the small intestine of NASH-vit. D rats. Significantly, plasma and portal endotoxin/TNFα levels, bacterial translocation to mesenteric lymph nodes, plasma DX-4000-FITC, fecal albumin-assessed intestinal hyper-permeability, over-expression of TNFα-related immune profiles in monocytes, inflammation of intestinal/mesenteric adipose tissue (MAT)/liver and hepatic steatosis were improved by chronic calcitriol treatment of NASH rats. Additionally, *in vitro* experiments with acute calcitriol co-incubation reversed NASH-V rat monocyte supernatant/TNFα-induced monolayer barrier dysfunction in caco-2 cells, cytokine release from MAT-derived adipocytes, and triglyceride synthesis by lean-V rat hepatocytes. Using *in vivo* and *in vitro* experiments, our study reported calcitriol signaling in the gut as well as in adipose tissue. Meanwhile, our study suggests that restoration of systemic and intestinal vitamin D deficiency using by chronic vitamin D treatment effectively reduces TNFα-mediated immunological abnormalities associated with the gut-adipose tissue-liver axis and hepatic steatosis in NASH rats.

## Introduction

Higher levels of plasma and intestinal lipopolysaccharide (LPS, also called endotoxin) are noted in nonalcoholic steatohepatitis (NASH) patients than in healthy subjects [1,2]. LPS is the main stimulator to induce tumor necrosis factor-α (TNFα) release from immune cells [1]. Compared to healthy controls, significantly high LPS-stimulated TNFα production is observed in cultured whole blood cells from NASH patients [2]. In NASH, increased TNFα can exacerbate intestinal inflammation and mucosal barrier disruption [3–5]. In the inflamed intestinal epithelium, TNFα produced from infiltrated immune cells further results in systemic/portal inflammation and endotoxemia [3–6]. So, NASH is characterized by remarkable intestinal hyper-permeability, epithelial tight junctions disruption and endotoxemia [5,7].

In obese animals, intestinal dysbiosis is associated with increased macrophage infiltration and high cytokine release by mesenteric adipose tissue (MAT), which is positioned near the intestine and is drained by the portal vein [8]. Additionally, intestinal hyper-permeability and MAT inflammation are involved in the pathogenesis of portal endotoxemia and hepatic steatosis [9,10]. Chronic intestinal/MAT inflammation and a disrupted intestinal barrier result in bacterial translocation and the progression of NAFLD to NASH [9,11,12]. Notably, in NASH, intestinal hyper-permeability, systemic/portal endotoxemia, and systemic/intestinal/MAT inflammation are initiated by TNFα-released from activated immune cells [2,4,5, 7,9,10]. So, anti-TNFα agents have the potential to simultaneously ameliorate the aforementioned abnormalities in NASH [9,10, 13,14].

In cultured human peripheral blood monocytes, 1,25-(OH)_2_D_3_, the hormonal form of vitamin D, is able to dose-dependently inhibit LPS-stimulated TNFα production [15]. Serum TNFα levels are negatively correlated with serum vitamin D concentrations in healthy women [16]. Among NASH patients, low serum vitamin D concentrations are closely associated with severe hepatic steatosis and inflammation [17]. Vitamin D receptor (VDR) is a nuclear receptor that mediates most of the known functions of vitamin D, including its anti-TNFα effects. Chronic mucosal inflammation and TNFα-induced down-regulation of gut epithelial VDR can be observed in cases of inflammatory bowel disease [18]. Through direct epithelial VDR up-regulation and indirect TNFα inhibition, vitamin D analog displays mucosal protection and anti-inflammatory effects [19].

Through histological evaluation in rat livers, vitamin D treatment from 6- weeks (time point for diet-induced NASH) to 12- weeks (time point for diet-induced fibrosis) after diet feeding has been found to prevent the progression of NASH to hepatic fibrosis [20]. With histological and gene expression measurements, depletion of vitamin D in a westernized diet exacerbates NAFLD through the activation of toll-like receptor (TLR) in NASH rat livers [21]. In addition to prevent the progression of NASH, vitamin D treatment reduced the serum free fatty acid/triglyceride levels, hepatic thiobarbituric acid-reactive substances (TBARS) levels, and hepatocyte apoptosis in rats [22]. Besides reducing hepatic steatosis, supplementation of vitamin D in the diet alleviates high-fat diet (HFD)-induced overweight and hyperinsulinemia by up-regulating hepatic lipolytic genes and adipose-tissue energy expenditure genes expressions in mice [23]. Especially in adipose tissue of obese rats, depletion of vitamin D in diet exacerbated HFD-increased adipose size by up-regulation of lipogenic/inflammatory genes and macrophage infiltration [24]. Further study reported that vitamin D treatment reduces hepatic triglyceride levels, hepatic nonalcoholic fatty liver disease activity score, and hepatic CD68/TGFβ1/αSMA expression, as well as decreases the levels of serum asparate aminotransferase and alanine aminotransferase in NASH rats [25]. Significantly, vitamin D treatment up-regulates nutrition sensing genes expression in adipose tissue of HFD-fed diabetic mice [26]. Chronic administration of vitamin D-enriched mushroom extracts reduces HFD-induced body fat accumulation, hepatic inflammation/steatosis (by histology and EchoMRI) and serum triglyceride/cytokine levels in mice [27].

To summarize the abovementioned studies [20–27], the therapeutic potential-associated with the anti-hepatic steatosis effects of vitamin D in the parallel abnormalities in circulation and the intestinal and adipose tissue of NASH animals have not been fully explored.

Accordingly, the present study aimed to evaluate the molecular mechanisms and effects of chronic vitamin D treatment on the aforementioned gut-adipose tissue-liver axis abnormalities using a rat model of diet-induced NASH.

## Materials and methods

### Animals

Male Sprague-Dawley (SD) rats (4-week old) were purchased from Charles River Japan, Inc. (Yokohama, Japan) and caged at 22°C on a 12-hour light-dark cycle with free access to water. Normal-chow-diet (NCD, Laboratory Autoclavable Rodent Diet 5010) or high-fat-diet (HFD, D12492) were given for 14 weeks to form the lean or NASH groups. From 4 to 14 weeks after NCD/HFD feeding, lean rats (Lean-V/lean-vit.D, n = 6 in each group) and NASH rats (NASH-V/NASH-vit.D, n = 9 in each group) received 10- weeks of either vehicle or 0.3 μg/kg/TIW of 1,25(OH)_2_D_3_ by gastric gavage.

This study was approved by the Animal Experiments Committee of Yang-Ming University and was performed according to the “Guide for the care and use of laboratory animals” prepared by the National Academy of Science, USA and the ARRIVE guidelines [28]. All efforts were made to minimize animal numbers necessary to produce reliable results and suffering was reduced by administering anesthetics (zoletil and xylocaine). At the end of the experiments, the rats were euthanized with 2–3 times the anesthetic dose of zoletil.

### Experimental design

Three days before the following experiments, *in vivo* intestinal permeability was measured in all rats. Subsequently, heparinized portal vein and whole-body blood was collected to separate peripheral blood mononuclear cells (PBMCs). Meanwhile, the liver, the intestine [doudenum, ileum, cecum and colon], mesenteric fats [the fat surrounding the gastrointestinal tract from the gastroesophageal sphincter to the end of the rectum], and the mesenteric lymph nodes [MLN, drained LNs from the terminal ileum, cecum and ascending colon] were collected by aseptic dissected. The macrophage numbers in the intestine and MAT were measured by flow cytometry. Primary monocytes (CD14^+^cells) were isolated from PBMCs and adipocytes were isolated from MAT.

In order to assess the *in vitro* effects of 1,25(OH)_2_D_3_ on TNFα and NASH-CM-induced cascades, the intestinal epithelial caco-2 cells and lean-V rat hepatocytes were cultured with/without different concentrations of 1,25(OH)_2_D_3_ [10^−11^, 10^−9^, 10^-7^M].

### Intestinal permeability

This measurement was based on the intestinal permeability to 4,000-Da fluorescent-dextran [DX-4000-fluorescein isothiocyanate (FITC), FD4000; Sigma-Aldrich, St. Louis, MO] by the analysis of time-dependent serum DX-4000-FITC concentration curves and area under curves (AUCs) [4,6,8]. For validation, the intestinal permeability was re-assessed by measurement of albumin content in the rat feces using ELISA kits (MyBioSource, Inc, San Diego, California, USA).

### Measurements of cytokines/cytokines receptors in PBMC-derived monocytes

After intestinal permeability measurement, heparinized-blood was collected from all rats for PBMCs (25–30×10^6^) isolation. PBMCs suspensions were depleted of neutrophils, NK cells, T-cells, and B-cells using immuno-magnetic sorting beads for magnetic cell sorting (MACS) by negative selection with Ly6G-biotin, CD56-biotin, CD3-biotin, and CD19-biotin antibodies. Further, primary rat monocytes (CD14^+^ cells) were isolated from above mentioned cell suspension as the CD14-biotin-positive, Ly6G/CD56/CD3/CD19-negative fraction separated by anti-CD14 mAb-coupled magnetic beads (Miltenyi Biotech, Bergisch Gladbach, Germany) through an MACS positive selection column. For flow cytometry analysis, monocytes (1 x 10^6^/ml/well) were fixed with 4% paraformaldehyde and permeabilized with 0.5% Triton X-100. Dead cells were then stained with propidium iodide (BD Biosciences) whereas live cells (monocyte) were stained, gated, and quantified for CD14/VDR,CD14/TNFα, CD14/NFκB,CD14/TNFRI and CD14/TNFRII double positive cells.

Monocytes were incubated for 20 h and cell free supernatants were harvested to measure TNFα, IL-6 and MCP-1 levels using ELISA kits (R&D, Minneapolis, MN). The supernatants of 48-hour-cultured NASH-V rat monocytes were used as conditioned medium (NASH-V-CM) to evaluate it effect on caco-2 cell monolayer integrity, lean-V rat adipocyte cytokine release and lean-V rat hepatocytes steatosis.

### Plasma cytokines/chemokines level

Plasma glucose, asparatate aminotransferase (AST), alanine aminotransferase (ALT), and triglyceride levels were measured using a standard auto SMAC analyzer (Roche Diagnostics Gmbh, ANNHEIM, Germany). Using pyrogen-free water (Lonza, Basel, Switzerland) and a pyrogen-free container, portal vein/plasma endotoxin, TNFα and LPS-binding protein (LBP), plasma/intestine calcitriol [1,25(OH)_2_D_3_] were measured using ToxinSensor Chromogenic LAL Endotoxin Assay Kit (GenScript USA Inc.) and cytokines/calcitriol ELISA Kits (R&D Systems INC., Minneapolis, MN). TG content in liver homogenate was measured by a TG Colorimetric Assay Kit (Cayman Chemical Company, Ann Arbor, MI, USA).

### Various intestinal and mesenteric adipose tissues (MAT) markers

Intestinal caspase-3 activity was determined. The protein and *mRNA* levels in the rat intestine, rat MAT-derived adipocytes, caco-2 cells, and rat hepatocytes were measured using appropriate antibodies and primers (S1 Table). For flow cytometry analysis, red blood cells (RBCs) present in 1 gram of intestinal and MAT homogenate were lysed using Pharm Lyse (BD Biosciences); the remaining cells (3×10^6^ cells/gram tissue) were suspended in PBS containing 2mM EDTA and exposed to FcBLOCK (BD Biosciences) for 20 minutes. To indentify macrophage infiltration (×10^3^/gram tissue) in tissues, the cells (3×10^5^ cells) were then labeled with FITC-conjugated F4/80 and PE-conjugated CD11b antibodies for quantification of CD11b(+)F4/80(+) cells by flow cytometry using a FACSalibur analyzer (BD Biosciences), whereas dead cells were identified using propidium iodide (BD Biosciences).

### Histological examination

NAFLD activity scores (NASs) were evaluated for rat liver samples. Duodenal tissue samples were used for immunohistochemical (IHC) studies of TNFα, TNFRI, and active caspase-7 expression.

### Bacterial translocation (BT) and fecal analysis

BT was defined as positivity of a bacterial culture in sterilized liquefied LMN. Three stool pellets (one per day) from each rat were pooled, dried and stored at 80°C to allow DNA extraction for quantification of the total number of bacterial cells, including intestinal *Lactobacillus* spp., *Bifidobacterium* spp. and *Bacteroides-Prevotella* spp.

### NASH-V-CM/TNFα-stimulated cytokines release from rat MAT-derived adipocytes

Lean-V and NASH-V adipocytes were isolated from rat MATs to measure time-dependent cytokine release [29]. Next, cytokines/cytokine receptor levels in the supernatant and *mRNA*s levels in the cell lysates of lean-V rat adipocytes treated with incremental concentration of 1,25(OH)_2_D_3_-treated were measured after stimulation with either TNFα or NASH-V-CM.

### *In vitro* effects of calcitriol on NASH-V-CM/TNFα-induced caco-2 cell monolayer barrier dysfunction

Differentiated caco-2 monolayer cells (5×10^5^) were treated with buffer, TNFα, or NASH-V-CM. Subsequently, 1,25(OH)_2_D_3_ was applied or was not to the apical and basolateral compartments for 48 hours. Next, barrier integrity was determined by measurement of the apical-to-basolateral flux of a fluorescent marker [fluorescein sulfonic acid (FSA; 200μg/mL; 478Da)] by the formula of FSA clearance (nl/h/cm^2^) = Fab/([FSA]a)×S) [30]. Fab is the apical-to-basolateral flux of FSA (light units/h), [FSA]a is the concentration at baseline (light units/nl) and S is the surface area (0.3cm^2^). Higher FSA represents more severe caco-2 monolayer mucosal dysfunction. Additionally, *mRNA/*protein expression levels in the caco-2 lysates were measured.

### *In vitro* effects of calcitriol on NASH-V-CM/TNFα-induced lipogenesis on lean-V rat hepatocytes

After isolation and standard preparation [29,31], lean-V rat hepatocytes (5×10^5^) were cultured with buffer, TNFα, or NASH-V-CM in the presence or absence of 1,25(OH)_2_D_3_ for 36 hours. Following this, oil red O stain-based measurement of intracellular TG accumulation was carried out. In parallel, expression of various *mRNA/*protein in corresponding cell lysates was measured.

#### Statistical analysis

Data were analyzed using Graphpad Prism 4 (GraphPad Software, San Diego, CA) and expressed as means±S.D. Statistical significance for each group was determined using one-way ANOVA with *post hoc* multiple comparisons being performed using the Newman-Keuls test. When the criteria for parametric testing were validated, Mann-Whitney U-tests were performed. *P* value less than 0.05 was considered significant.

## Results

**Fig 1:**
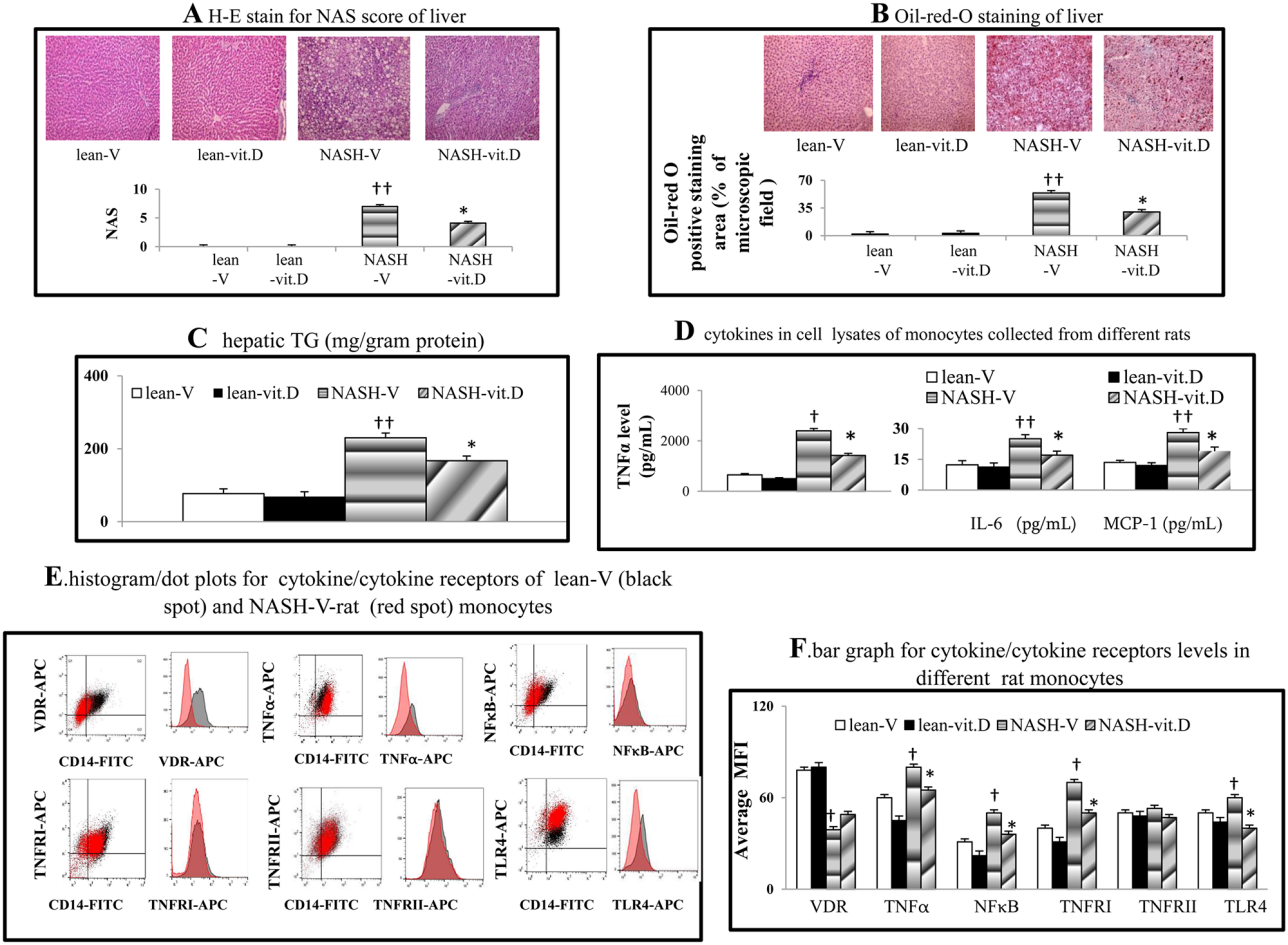
Chronic calcitriol treatment ameliorates hepatic steatosis and improved inflammatory profiles of monocytes. H-E (a) and O-red oil staining (b) for hepatic steatosis. (c) hepatic triglyceride content and (d) cytokines levels in the cell lysates of various rat monocytes. (e) A representative flow cytometric histogram/dot plots of the cytokines/cytokine receptors of NASH-V-rat monocytes. (f) A bar graph of the flow cytometry-assessed cytokines/cytokine receptors of monocytes from various rats. ^†^*P*<0.05, ^††^ *P*<0.01 *vs*. lean-V group; *P< 0.05, **P<0.01 *vs*. NASH-V group.

Notably, 14 weeks of high-fat diet (HFD) feeding induced typical NASH histology as well as high ALT, AST, fasting glucose and triglyceride levels in NASH-V rats (Fig 1A, Table 1). Significantly, the HE stain-assessed NAS scores, O-red-oil stain-assessed hepatic steatosis, hepatic triglyceride levels, body weight, and liver weight in NASH-V rats were higher than those in the lean-V rats (Fig 1A–1C, Table 1).

**Table 1:** Basal characteristics of all rats.

	Lean-V (n = 6)	Lean-vit. D (n = 6)	NASH-V (n = 9)	NASH-vit.D (n = 9)
Body weight (gram)	399±17	406±19	508±49^††^	462±30*
Liver weight (gram)	15.7±1.3	17.9±1.8	37.1±1.2^†^	29.3±2.5*
[fasting glucose] (mg/dL)	107.5±4.8	98.7±10.5	227.3 ±8.1^†^	165.4±9.2*
[Triglyceride] (mg/dL)	59±4	50±10	199±18^††^	95±8**
[aspartate aminotransferase] (AST, U/L)	49.6±5.1	48.5±4.3	111.4±3.5^†^	80.7± 8.6*
[alanine aminotransferase] (ALT, U/L)	45.8±6.2	35.9±4.7	91.7±6.2^††^	70.7±3.9**

Data were expressed as mean ±SD;

^†^*P*<0.05,

^††^ *P*<0.01 vs. lean-V rat's data;

* *P* <0.05,

**P<0.01 *vs*. NASH-V rat's data.

### Correction of the hypo-vitaminemia D inhibits systemic/portal endotoxemia and systemic inflammatory profiles

Compared to lean-V rats, NASH-V rats were found to have higher levels of plasma TNFα, LBP, and plasma/portal endotoxin, as well as lower levels of plasma/intestinal calcitriol (Table 2). Compared to the NASH-V group, restoration of plasma calcitriol levels in NASH-vit.D rats by 10 weeks of calcitriol treatment was accompanied by the suppression of plasma and portal endotoxin levels as well as the reduction of LBP and TNFα levels (Table 2). Higher TNFα, TNFRI, NFκB, and TLR4 expression in NASH-V rat monocytes than in the lean-V group was observed and this was accompanied by lower vitamin D receptor (VDR) expression (Fig 1D–1F). Significantly, 10 weeks of calcitriol treatment suppressed the TNFα, TNFRI, NFκB and TLR4 expression and normalized VDR expression in the NASH rat monocytes (Fig 1D–1F). Nonetheless, no significant difference in the aforementioned markers was found when lean-V and lean-vit.D groups were compared.

**Table 2:** Various pathogenic markers in all rats.

	Lean-V (n = 6)	Lean-vit. D (n = 6)	NASH-V (n = 9)	NASH-vit.D (n = 9)
[Calcitriol, 1,25(OH)_2_D_3_, pg/mL]	12.1±0.4	14.8±1.3	4.6±1.1^††^	11.9±2.1**
[Endotoxin, EU/mL]	3.8±0.4	3.2±0.2	17.8±2.3^††^	8.8±1.1**
Portal venous endotoxin levels [EU/mL]	4.1±0.08	3.7±0.05	19.5±1.1^††^	9.4±0.9**
[TNFα, pg/mL]	4.8±1.1	4.1± 0.7	22.1±3.5^††^	13.9±2.1*
[LPS binding protein, LBP, ng/mL]	309±13	252±28	4239±547^††^	2520±438**
Bacterial-translocation (BT rate, %) positive culture of mesenteric lymph node (MLN)	0	0	6/9 (67%)^††^	3/9(33%) *
Intestinal calcitriol [1,25(OH)_2_D_3_, pg/gram]	80±11	82±7	41±8^††^	69±10 *
Intestinal caspase-3 activity (fold changes compared to lean-V)	1	0.9±0.2	4.3±0.5 ^†^	2.9±0.4*
Hepatic TNFα levels (pg/mg protein)	25±1	22±4	66±3^†^	42±7*
Hepatic MCP-1 levels (pg/mg protein)	220±11	198±23	519±16^††^	403±38*
Hepatic IL-6 levels (pg/mg protein)	27±3	21±8	58±17^†^	42±19*

Data were expressed as mean ±SD;

^†^*P*<0.05,

^††^ *P*<0.01 vs. lean-V rat's data;

* *P* <0.05,

**P<0.01 *vs*. NASH-V rat's data.

### Chronic calcitriol treatment suppresses intestinal hyper-permeability and intestinal pathogenic signals in NASH rats

**Fig 2:**
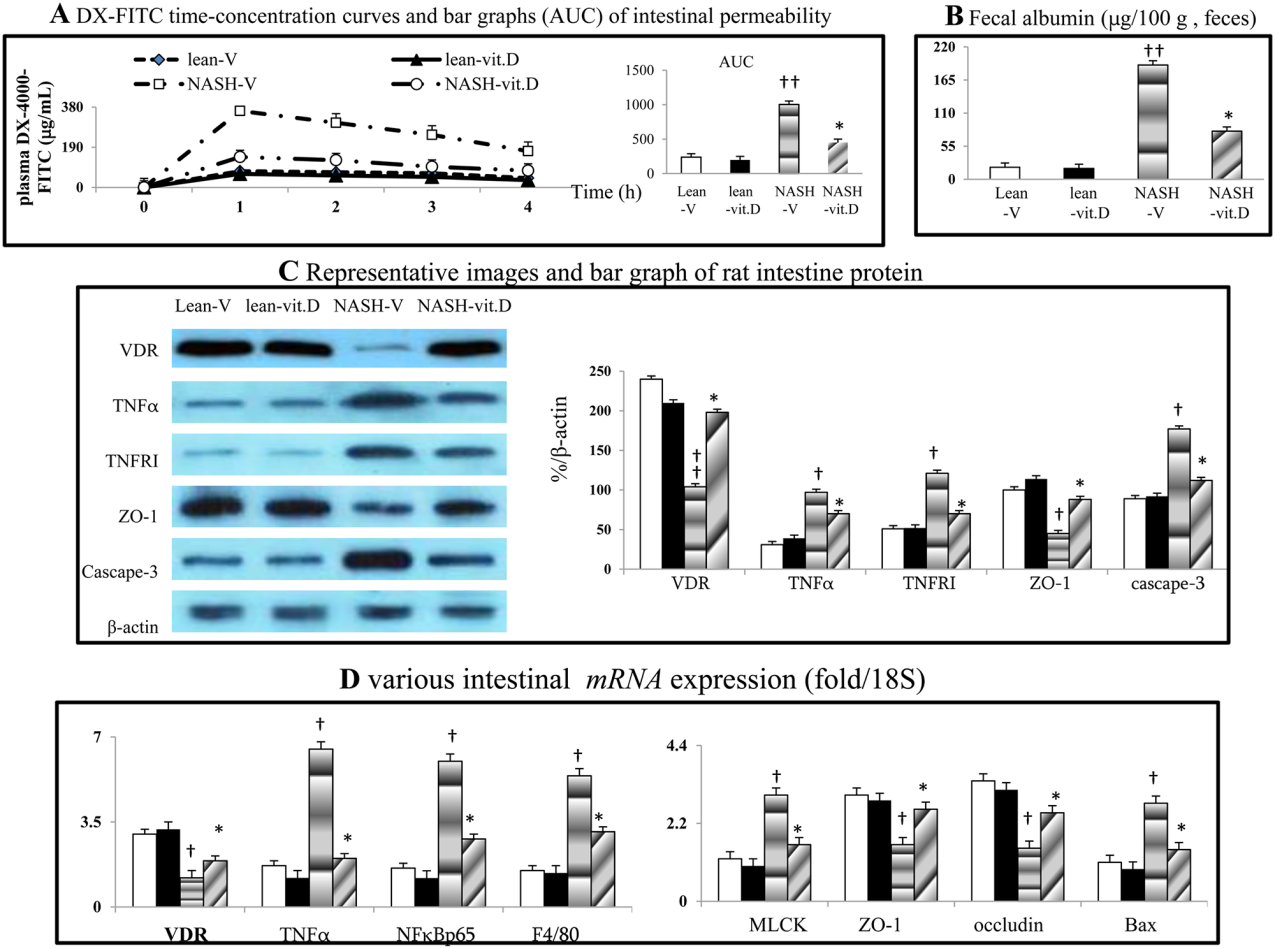
Chronic calcitriol treatment normalizes intestinal VDR expression and improves intestinal hyper-permeability in NASH rats. DX-4000 FITC-based (a) and fecal albumin-based (b) assessment of intestinal permeability. The expression of various proteins (c) and *mRNAs* (d) in intestines from different groups of rat; AUC: area under curves; ^†^*P*<0.05, ^††^ *P*<0.01 *vs*. lean-V group; *P< 0.05, **P<0.01 *vs*. NASH-V group.

**Fig 3:**
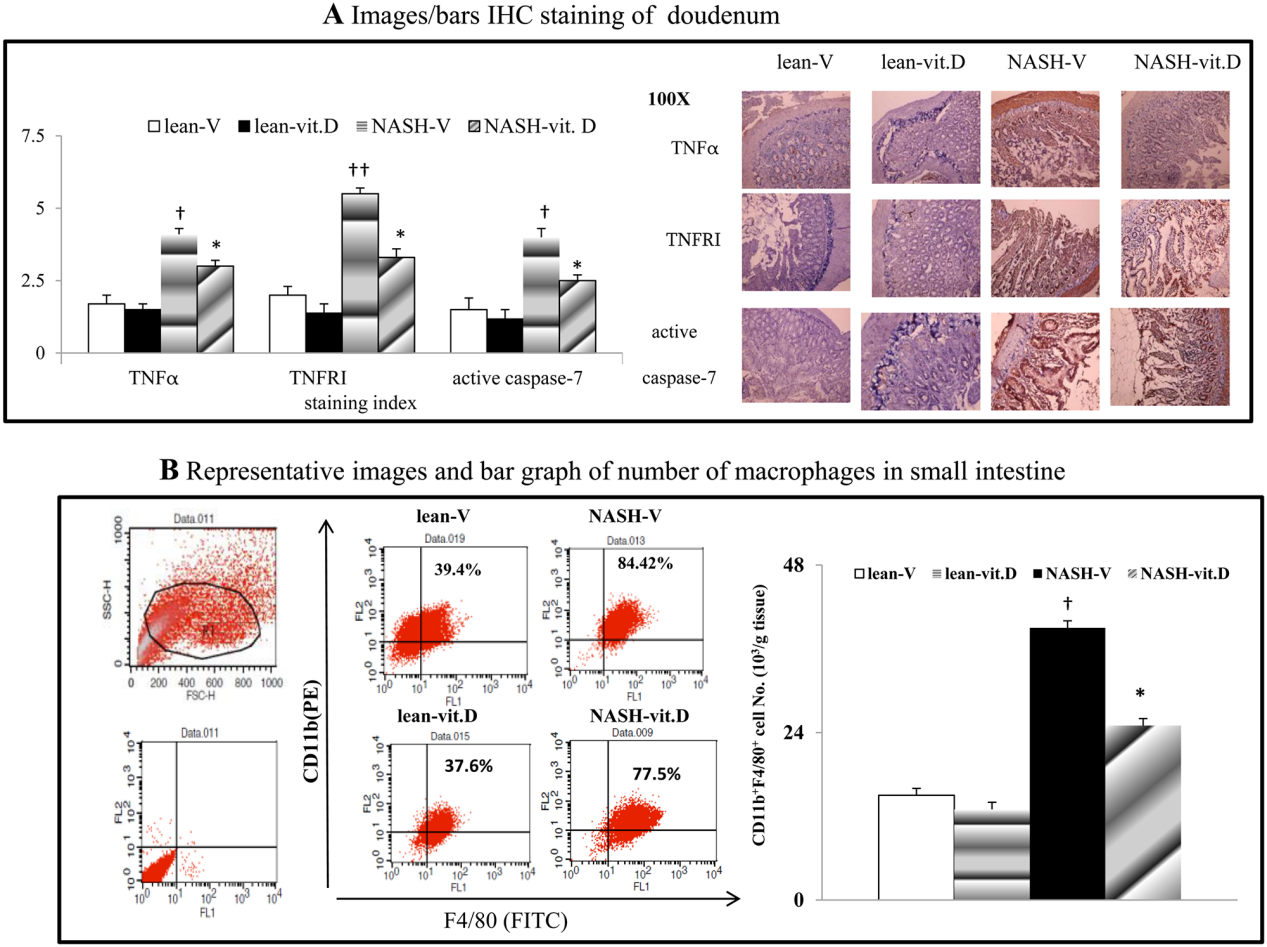
Chronic calcitriol treatment improved intestinal inflammation in NASH rats. The expression of various proteins (a) in the intestines from various different groups of rat and the flow-cytometry-based analysis of macrophage infiltration in the same rat (b) small intestine. ^†^*P*<0.05, ^††^ *P*<0.01 *vs*. lean-V group; *P< 0.05, **P<0.01 *vs*. NASH-V group.

In NASH-V rats, intestinal hyper-permeability (DX-4000-FITC and fecal albumin-based assays) and down-regulated tight-junction proteins (ZO-1 and occludin) expressions were associated with the decreased intestinal VDR and increased intestinal TNFα, TNFRI, caspase-7, Bax and MLCK levels (Fig 2A–2D). Furthermore, the aforementioned intestinal abnormalities were significantly suppressed in NASH-V rats (Figs 2 and 3A).

Significantly, intestinal hyper-permeability was associated with an abundance of *Bacteroides-Prevotella* and a reduction in *Lactobacillus* (↓89%) and *Bifidobacterium* (↓79%) numbers in NASH-V rats. However, the improvement in intestinal hyperpermeability in NASH-vit. D rats was not accompanied by the reversal of rat gut microbiota changes (Table 3).

**Table 3:** Qantification of total number of bacterial cells of the intestinal flora in cecal content.

	Lean-V	Lean-vit. D	NASH-V	NASH-vit.D
*Lactobacillus* spp. (cells/g cecal content)	7.1×10^8^±2.9×10^8^	8.5×10^8^±3.6×10^8^	2.3×10^7^±0.49×10^7^^†^	2.9×10^7^±0.86×10^7^
*Bifidobacterium* spp. (cells/g cecal content)	5.3×10^6^±1.4×10^6^	6.8×10^6^±0.9×10^6^	1.4×10^4^±0.57×10^4^^††^	6.6×10^4^±0.89×10^4^
*Bacteroides-Prevotella* spp. (cells/g cecal content)	4.9×10^8^±0.43×10^8^	1.1×10^8^±0.23×10^8^	2.9×10^9^±0.55×10^9^^†^	1.9×10^8^±0.27×10^8^

Data were expressed as mean ±SD;

^†^*P*<0.05,

^††^ *P*<0.01 vs. lean-V rat's cecal content.

### Intestinal hyper-permeability parallels to the portal endotoxemia and tissue inflammation in NASH rats

**Fig 4:**
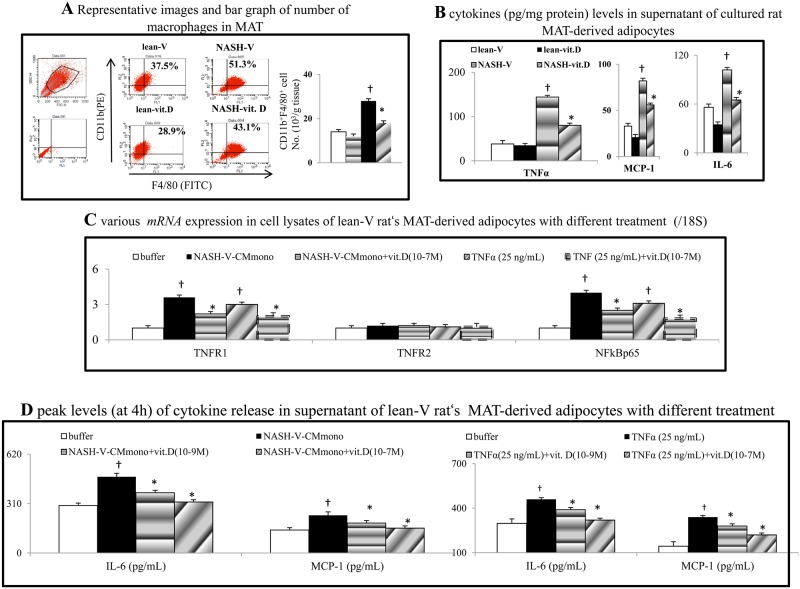
Chronic calcitriol treatment suppresses mesenteric adipose tissue (MAT) inflammation of NASH rats. (a) A flow-cytometry-based analysis of macrophage infiltration in rat MAT. (b) The cytokines levels in the supernatant of MAT-derived adipocytes collected from different groups of rats. (c) The expression levels of *mRNA*s in the cell lysates of lean-V rat adipocytes after various treatments. (d) The peak levels of cytokine releases in the supernatant of NASH-V rat adipocytes after various treatments; †*P*<0.05, †† *P*<0.01 *vs*. lean-V/buffer group; *P< 0.05, **P<0.01 vs. NASH-V-CM/TNFα group.

Notably, the intestinal hyper-permeability was associated with portal endotoxemia and increased hepatic/MAT/intestinal macrophage infiltration, inflammation and corresponding cytokines levels (IL-6, MCP-1, *F4/80*, TNFα, *NFκBp65*, TNFRI and TLR4) in NASH-V rats (Tables 1 and 2, Figs 2C, 2D, 3 and 4).

### Chronic calcitriol treatment suppresses MAT inflammation and reduces the inflammatory profiles of NASH-V rat adipocytes

Compared to the lean-V group, a significant increase in the number of infiltrated macrophages in MAT was noted in the NASH-V group (Fig 4A). In addition, the releases of TNFα, MCP-1 and IL-6 from MAT-derived adipocytes was higher in the NASH-vit. D group than that in the NASH-V group (Fig 4B). In lean-V rat adipocytes cultures, NASH-V-CM and TNFα induce the release of aforementioned cytokines and up-regulated the *TNFR1*/*NFκB* expressions in cell lysates, which were dose-dependently abolished by vitamin D co-incubation (Fig 4C and 4D).

### Acute calcitriol incubation prevents NASH-V-CM/TNFα-induced caco-2 monolayer mucosal dysfunction

**Fig 5:**
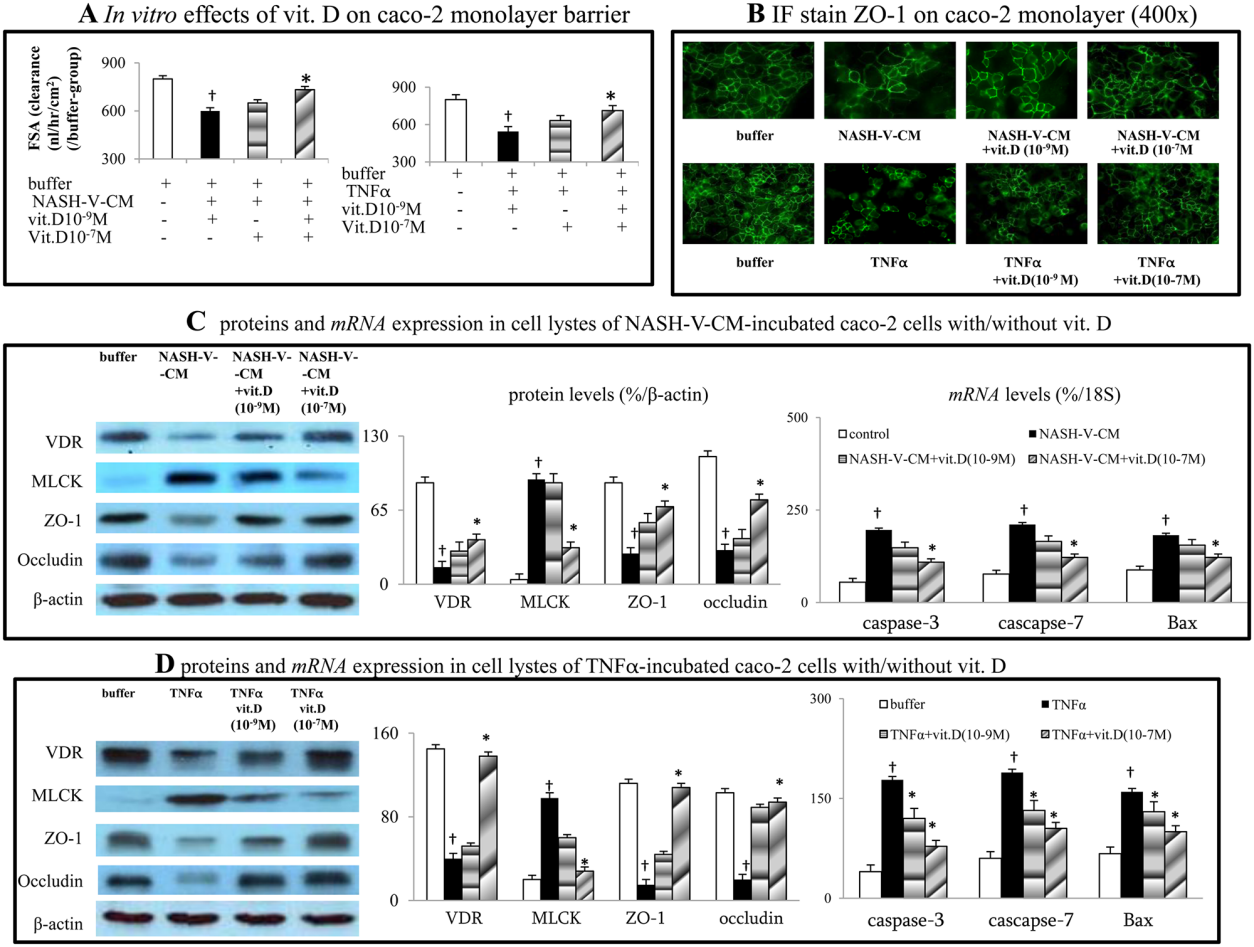
*In vitro* effects of calcitriol on the NASH-V-CM and TNFα-induced mucosal dysfunction in caco-2 cells. (a,b) The *in vitro* effects of various treatments on caco-2 monolayer mucosal dysfunction and IF-stained ZO-1 expression. (c,d) protein and *mRNA* levels in the cell lysates of caco-2 monolayer cells after various treatments. ^†^*P*<0.05, ^††^ *P*<0.01 *vs*. lean-V group; *P< 0.05, **P<0.01 *vs*. NASH-V-CM group.

Fig 5A and 5B revealed that NASH-V-CM and TNFα induced barrier dysfunction and caused a decrease in the IF-evaluated ZO-1 stained positive area of caco-2 monolayers. These changes were accompanied by down-regulation of VDR, ZO-1 and occludin expression, as well as up-regulation of *caspase-3*, *cascapse-7*, *Bax*, and MLCK expression in caco-2 cell lysates (Fig 5C and 5D). Dose-dependently, incubation with vitamin D normalized the aforementioned NASH-V-CM and TNFα-induced changes in the caco-2 cells culture system (Fig 5).

### Acute calcitriol incubation prevents NASH-V-CM/TNFα-induced lean-V rat hepatocytes' lipogenesis

**Fig 6:**
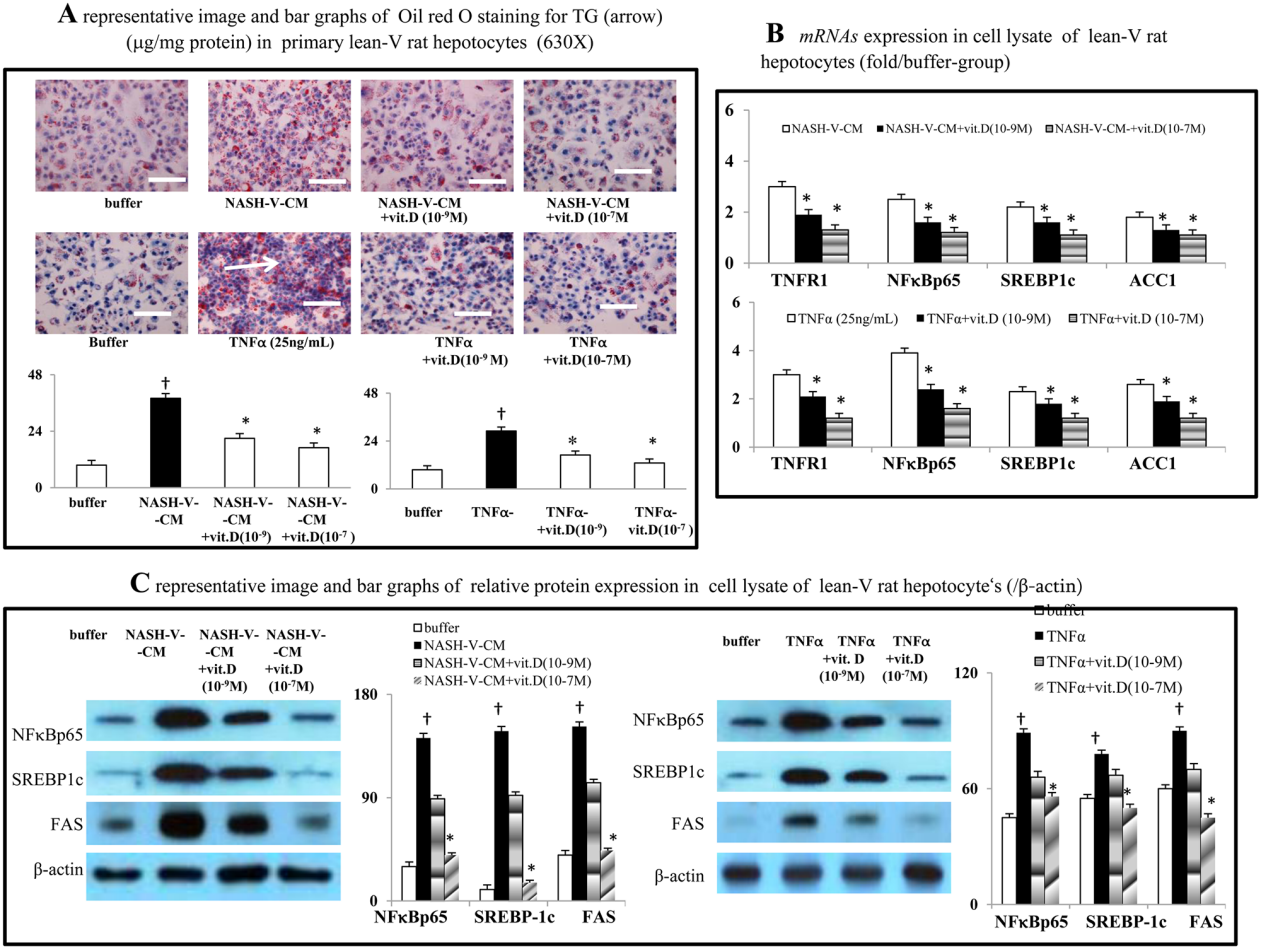
*In vitro* effects of calcitriol on the NASH-V-CM and TNFα-induced lipogenesis in lean-V rat hepatocytes. (a) representative micrographs of intracellular lipogenesis in lean-V rat hepatocytes after various different treatments. (b,c) The cytokines levels, lipogenic protein/*mRNA* levels in the cell lysates of lean-V rat hepatocytes after various treatments. ^†^*P*<0.05, ^††^ *P*<0.01 *vs*. buffer-group; *P< 0.05, **P<0.01 *vs*. NASH-V-CM-group/TNFα-group.

Compared to the buffer group, NASH-V-CM and TNFα (25ng/mL) significantly increased the intracellular triglyceride content of lean-V rat hepatocytes (Fig 6A). Furthermore, the HFD-V-CM and TNFα-induced lean-V rat hepatocyte lipogenesis was accompanied by up-regulation of *TNFR1*/NFκBp65 as well as lipogenic signals in the cell lysates (Fig 6B and 6C). Notably, incubation with vitamin D dose-dependently reversed the aforementioned NASH-V-CM and TNFα-induced changes in the lean-V rat hepatocyte culture system (Fig 6A–6C).

## Discussion

Increased TNFα-TNFR1 gene expression and elevated soluble TNFα levels are well-recognized pathogenic factors for NASH development [32,33]. Vitamin D can suppress TNF-α and IL-6 production by activated-monocytes in type 2 diabetic patients [34]. In cultured peritoneal macrophages of patients undergoing peritoneal dialysis, vitamin D dose-dependently inhibit LPS-stimulated TNFα release [35]. Our study reveals that the cytokines (TNF-α, IL-6, and MCP-1) released from NASH-V rat monocytes is higher than those in the lean-V group. Significantly, in our study, chronic vitamin D treatment inhibited the cytokines levels in monocytes of NASH-vit. D rats compared to those in NASH-V rats. TNFα-mediated inflammatory responses are mainly mediated by TNFR1 [36]. So, it is reasonable to observe that TNFα-related effects were found to be mediated by the TNFR1-NFκB pathway in monocytes and the tissues of NASH-V rats.

Vitamin D and vitamin D receptors (VDRs) are important regulators of intestinal inflammation [18,19,31,37]. In particular, vitamin D and VDR deficiency exacerbates experimental inflammatory bowel disease (IBD) [18,19,37]. It is reported that VDR gene-knocknout mice develop severe intestinal inflammation in experimental models of IBD [37–39]. *In vitro* studies have shown that vitamin D protects against dextran sodium sulfate (DSS)-induced disruption of intestinal epithelial tight junctions [40,41]. In our NASH rats, the intestine VDR expression was negatively correlated with the animals' intestinal TNFα levels, intestinal hyper-permeability and tight junction protein expression and these could be corrected by chronic vitamin D treatment.

Binding of TNFα to TNFR, which activates myosin light chain kinase (MLCK) and NFκB in intestinal epithelial cells can result in epithelial mucosal barrier dysfunction [40–42]. Intestinal MLCK over-expression has been reported in patients with ulcerative colitis and Crohn’s disease [43]. Up-regulated MLCK and NFκB can induce apoptosis and rearrangement of tight junction proteins, including occludin and ZO-1 [40,41–43]. In our NASH rats receiving chronic vitamin D treatment, down-regulation of intestinal TNFα-TNFR1-NFκB signals prevented intestinal apoptotic activity and preserved the integrity of the intestinal mucosal barrier by decreasing MLCK expression and normalizing tight-junction protein expression. In our caco-2 cell system, TNFα and the supernatant of cultured NASH-V rat monocytes could induce monolayer mucosal dysfunction and the corresponding pathogenic signals, which could be inhibited by vitamin D co-incubation.

In experimental colitis models, 1,25(OH)_2_vitamin D_3_ has been found to suppress intestinal mucosal injury, decrease intestinal inflammation and maintain the integrity of the intestinal mucosal barrier [38,39]. A negative correlation has been noted between serum vitamin D and bacterial-translocation in HIV/hepattis C virus co-infected patients [44]. High LBP levels and MLN positive culture rates are representative markers for increased bacterial-translocation [44,45]. In our NASH rats receiving vitamin D treatment, normalization of plasma/tissue calcitriol and VDR levels was accompanied by a decrease in the MLN positive culture rate and in plasma LBP levels. In addition to bacterial-translocation, alteration in gut microbiota is known to be involved in the progression of NASH [3,13,14]. Modulation of gut microbiota ameliorates obesity-associated impaired intestinal mucosal intergrity and bacterial-translocation in rats [13,14]. In our study, the lack of an effect of chronic vitamin D treatment on the gut microbiota indicates that the benefits of chronic vitamin D treatment in our NASH-vit.D rats involved other mechanisms.

In our lean rat mesenteric adipose tissue (MAT)-derived adipocytes, TNFα and the supernatant of cultured NASH-V rat monocytes induced cytokines release, which could be inhibited by vitamin D co-incubation. The blood and lymphatic vessels draining the gut are embedded in MAT [46]. In NASH animals with endotoxemia, there is a positive regulatory loop between intestinal mucosal barrier dysfunction and MAT inflammation [6,8]. In our NASH rats, vitamin D treatment-related correction of intestinal mucosal barrier dysfunction was accompanied by an improvement in MAT inflammation and a reduction in endotoxemia. In fact, a causal link has been reported between the HFD-induced gut inflammation and the activated inflammatory profile in MAT adjacent to the inflamed intestine [10]. In inflamed adipose tissue, vitamin D can suppress the TNFα and NFκB-mediated cytokines release [47,48]. In NAFLD patients, vitamin D deficiency has been reported to increase the risk of NASH via activation of NFκB signals [17,49]. So, it is reasonable to observe in our NASH rats that macrophage infiltration, inflammation, and TNFα-NFκB-mediated cytokines release in gut and adipose tissues were simultaneously inhibited by chronic vitamin D treatment.

Blood from the portal vein drains to the liver and thus, the liver is exposed to relatively high concentrations of TNFα and IL-6 released by the inflammed gut and MAT [50]. Both *in vivo* and *in vitro* studies have reported that TNFα and IL-6 can exacerbate hepatic steatosis [51–53]. TNFα can activate NFκB and stimulate IL-6 production from hepatocytes [54]. Vitamin D treatment suppresses hepatic lipogenesis by down-regulation of lipogenic signals [23,25,55]. In our study, chronic vitamin D treatment decrease hepatic steatosis by suppressing the levels of TNFα, NFκB and IL-6 in NASH rat livers. Additionally, in an *in vitro* study, the suppression of TNFα-TNFR1-NFκB signaling and corresponding lipogenesis by vitamin D co-incubation was observed in lean rat hepatocytes.

In conclusion, our study suggests that systemic/portal endotoxemia, intestinal inflammation, intestinal hyper-permeability, together with up-regulation of TNFα-mediated signaling contribute to down-regulation of VDR expression in monocytes as well as within the intestine of NASH rats. Furthermore, intestinal hyper-permeability exacerbates bacterial translocation to mesenteric lymph nodes and mesenteric adipose tissue inflammation; this subsequently leads to TNFα-TNFR1-NFκB-mediated hepatic steatosis in NASH rats. Intriguingly, the intestinal VDR deficiencies as well as the related TNFα-TNFR1-NFκB-mediated gut/adipose/liver abnormalities can be effectively attenuated by chronic calcitriol treatment in NASH animals. So, in addition to diet control and exercise, chronic use of vitamin D may be a promising strategy for the improvement of NASH.

## Supporting information

S1 Table Primer of rat gene used for quantitative realtime PCR analysis. (DOCX)

S1 Fig Chronic calcitriol treatment dose-dependently inhibits hepatic steatosis and serum/intestinal TNFα levels of NASH-V rats. (TIF)

S1 File Supplement materials and methods. (DOCX)

S2 File Laboratory protocols of the procedure of isolation of adipocytes from rat mesenteric adipose tissue (MAT) in protocols.io. (DOCX)

S3 File Data of this study. (XLSX)
