# Supplementary material for: Chronic calcitriol supplementation improves the inflammatory profiles of circulating monocytes and the associated intestinal/adipose tissue alteration in a diet-induced steatohepatitis rat model
Source: PLoS One. 2018 Apr 23;13(4):e0194867. doi: 10.1371/journal.pone.0194867 (PMC5912737; doi:10.1371/journal.pone.0194867)
Supplement: S1 Table — (DOCX) [file pone.0194867.s001.docx]

**Supplement Table 1. primer of rat gene used for quantitative realtime PCR analysis.**

| Gene name | Primers sequences |
| --- | --- |
| *VDR* | For:5′- GCCCCTCATAAAGTTCCAGGTG; Rev′- GGATAGGCGG  TCCTGAATGG -3′ |
| *TNFα* | For:5′-GCTCACAATGTCTGTGCTTAGAG-3′; Rev:5′-GCAGTAG  CCACAGCTCCAG-3′ |
| *TNFR1* | For:5′- TGACCCTCTCCTCTACGGA-3′; Rev:5′- CCATCCACCA  CAGCATACA-3′ |
| *TNFR2* | For:5′- TAGGACTGGCGAACTGCTT -3′; Rev:5′- AACTGGGTG  CTGTGGTCAAT-3′ |
| *NFκBp65* | For:5′- GGCCTCATCCACATGAACTT -3′; Rev:5′- CACTGTCACCT  GGAAGCAGA-3′ |
| *F4/80* | For:5′-TGACAACCAGACGGCTTGTG-3; ′Rev:5′- GCAGGCGAG  GAAAAG ATA GTGT -3′ |
| *MLCK* | For: 5′-AAT GGT GTT GCT GGA GAT CGA GGT -3′;Rev: 5′- CTCAAAGTTACCACCGCTGCTG-3′ |
| *ZO-1* | For: 5′- CGGGACTGTTGGTATTGGCTAGA -3′;Rev: 5′- GGCCAG  GGCCATAGTAAAGTTTG-3′ |
| *Occludin* | For: 5′- TCCTATAAATCCACGCCGGTTC -3′;Rev: 5′- CTCAAAG  TTACCACCGCTGCTG-3′ |
| *Bax* | For: 5′- GGCGATGAACTGGACAAC -3′;Rev: 5′- GTGAGTGAGG  CAGTGAGGA-3′ |
| *Caspase-3* | For:5′- GGTATTGAGACAGACAGTGG -3′; Rev:5′- CATGGGATC  TGTTTCTTTGC -3′ |
| *Caspase-7* | For:5′- TGAGCCACGGAGAAGAGAAT -3′; Rev:5′- TTTGCTTAC  TCCACGGTTCC -3′ |
| *SREBP-1c* | Forward :5''-GGCACTAAGTGCCCTCAACCT-3′;′Reverse: 5′-GCC  ACATAGATCTCTGCCAGTGT-3′ |
| *ACC1* | For: 5′-GCCATCCGGTTTGTTGTCA -3′; Rev: 5′-GGATACCTG  CAGTTTGAGCCA-3′ |
| *18S* | For:5′-GTAACCCGTTGAACCCCATT-3′; Rev:5′-CCATCCAA  TCGGTAGTAGCG-3 |

TNFα, Tumor necrosis factor-alpha; MLKC: myosin light chain kinase; Bax: apoptotic promoter
