# Supplementary figures and images for: Chronic calcitriol supplementation improves the inflammatory profiles of circulating monocytes and the associated intestinal/adipose tissue alteration in a diet-induced steatohepatitis rat model
Source: PLoS One. 2018 Apr 23;13(4):e0194867. doi: 10.1371/journal.pone.0194867 (PMC5912737; doi:10.1371/journal.pone.0194867)

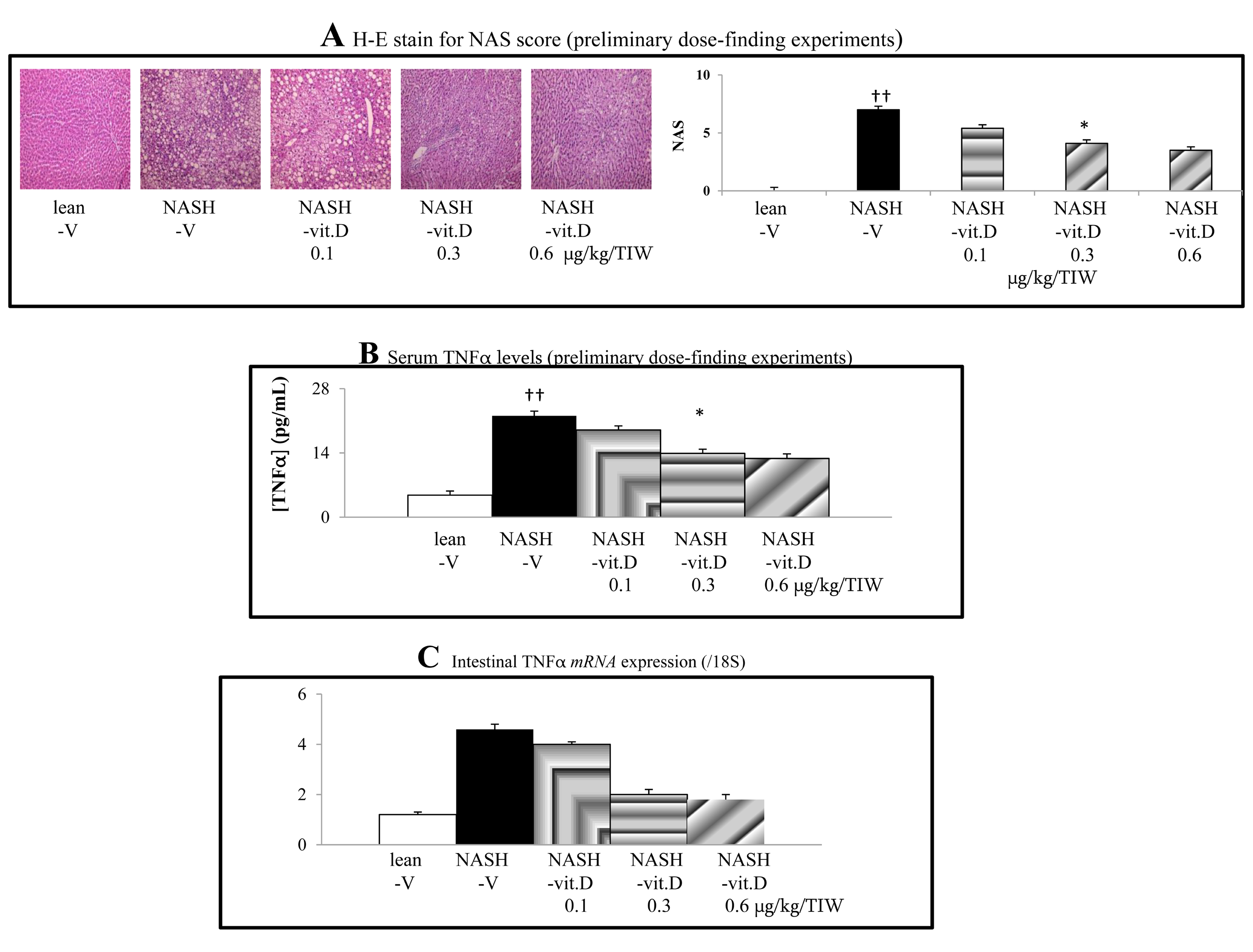

Supplement: S1 Fig — (TIF) [file pone.0194867.s002.tif]
