# Supplementary material for: Chronic calcitriol supplementation improves the inflammatory profiles of circulating monocytes and the associated intestinal/adipose tissue alteration in a diet-induced steatohepatitis rat model
Source: PLoS One. 2018 Apr 23;13(4):e0194867. doi: 10.1371/journal.pone.0194867 (PMC5912737; doi:10.1371/journal.pone.0194867)
Supplement: S1 File — (DOCX) [file pone.0194867.s003.docx]

**Supplement materials and methods**

**Animals**

In NAFLD animals, the time-point for development of increased circulating TNFα-related gut-adipose tissue-liver axis abnormalities is 4-week after HFD feeding [1,2]. Recent study had revealed that orally administration of 1α-hydroxy-cholecalcifrol (1α(OH)D_3_) three times/week for 6-week dose dependently (0.1 or 0.4 μg/kg) ameliorated NASH in rats [3].

So, to evaluate the effects of vitamin D treatment on the TNFα-mediated abnormalities, four groups of rats were included in this dose-finding preliminary experiments (n=3) to receive 10-week of 0.1, 0.3 or 0.6 μg/kg three times/week (TIW) of calcitriol [1,25(OH)_2_D_3_] (NASH-vit.D-0.1, -0.3, -0.6) or vehicle (NASH-V) by oral gavages from 4-week (SS) until 14-week after HFD feeding. As a ligand for the vitamin D receptor (VDR), calcitriol [1,25(OH)_2_D_3_] is the major circulating form of vitamin D_3_. In comparison with NASH-V rats, plasma/intestinal TNFα level/*mRNA* and hepatic NAFLD activity score (NAS) were not significantly changed in NASH-vit.D-0.1 rats (supplement Fig. 1A-C). However, the plasma/intestinal TNFα level/*mRNA* and hepatic NAS were significantly decreased in NASH-vit.D-0.3 rats than in NASH-V rats. Further, the plasma/intestinal TNFα level/*mRNA* and hepatic NAS were not different between NASH-vit.D-0.3 and NASH-vit.D-0.5 rats.

**Intestinal permeability**

Overnight-fasted rats were gavaged with DX-4000-FITC (600 mg/kg body wt, 125 mg/ml). After 0 h, 1 h, 2h, 3h and 4 h, 500μL of blood was collected from the tip of the tail vein. The blood was centrifuged at 4°C, 12,000g, for 3 min. Plasma was diluted in an equal volume of PBS (pH 7.4) and analyzed for FITC-dextran concentration with a ﬂuorescence spectrophotometer (HTS-7000 Plus-plate-reader; Perkin Elmer, Wellesley, MA) at the excitation wavelength of 485 nm and the emission wavelength of 535 nm. Standard curves for calculating the DX-4000-FITC concentration in the samples were obtained by diluting FITC-dextran in non-treated plasma diluted with PBS (1:2 [vol/vol]). The intestinal permeability was assessed additionally by measuring the albumin content in the feces by ELISA kit. Feces was diluted in dilution buffer (100mg/ml) and analyzed following the manufacturer’s instructions.

**Measurement of endotoxin, LBP and calcitriol levels**

Briefly, 0.1 ml plasma and portal vein samples were incubated with 0.1 ml Limulus amebocyte lysate (LAL) at 37°C for 45 min. After several subsequent reactions, the samples were read spectrophotometrically at 545 nm. The plasma endotoxin levels were calculated against a standard curve of endotoxin (E. coli 0113:H10) concentrations of 0.1, 0.04, 0.02, 0.01 and 0.005 EU/ml. Plasma calcitriol (1,25(OH)2D3) levels was measured by ELISA kits with the linear detection range was 0.08 to 20 mg/dl.

**Various intestinal markers**

Caspase-3 activity was calorimetrically assayed using R &D systems kit as described by the manufacturer. Briefly, an aliquot of the homogenate supernatant was incubated with the labeled substrate DEVD-pNA (acetyl-Asp-Glu-Val-Asp p-nitroanilide). The cleavage of the peptide by the caspase releases the chromophore pNA, which was read at 405 nm using Biochrom Asys microplate reader, UK. According to the manufacturer’s instructions, the results were expressed as fold of caspase-3 activity of lean-V group.

**Histological examination**

Hepatic NAFLD activity score (NAS) is evaluated in all rat livers and compared between groups. NAS score is the summative scores of steatosis (0-3), lobular inflammation (0-2), hepatocellular ballooning (0-2) and fibrosis (0-4). It had been reported that the NAS score around 3-4 indicate severe HS and ≧ 5 corresponds with NASH. Moreover, doudenal tissue samples were fixed in 4% buffered formalin and embedded in paraffin. Next, sections were incubated with pre-diluted TNFα, TNFRI and active caspase-7 antibodies or rabbit pre-immune serum (negative controls), followed by avidin-biotin-peroxidase (Vector, urlingame, CA) for the immunohistochemical (IHC) studies.

**Fecal analyses**

The QIAamp DNA Stool Minikit (Qiagen) was used to extract DNA from stool sample according to the manufacturer’s instructions for Quantitative RT-PCR quantiﬁcation of microbial cecal content. The primers and probes used to detect *Lactobacillus* spp., *Biﬁdobacterium* spp. and *Bacteroides-Prevotella* spp. were based on 16S rRNA gene sequences. The PCR ampliﬁcation reactions were carried out as follows: 2 min at 50°C, 10 min at 95°C, followed by 45 cycles of 15 s at 95°C and 1 min at 60°C. Detection was carried out on an ABI Prism 7900 sequence detection system (Applied Biosystems, Foster City, CA). Each assay was performed in duplicate in the same run. The cycle threshold of each sample was then compared with a standard curve made by diluting genomic DNA (10-fold serial dilution) from cultures. Cell counts before DNA extraction were determined with the Neubauer hemocytometer. To determine the sensitivity and speciﬁcity of the assays, the PCR assays were conﬁrmed using a set of intestinal bacterial species as controls. Group-speciﬁc primers based on 16 S rDNA sequences PCR assay are forward *Biﬁdobacterium*, cgcgtcyggtgtgaag; reverse *Biﬁdobacterium*, ccccacatccagcatcca; BHQ-1-biﬁdo,aacaggattagataccc; forward *Lactobacillus*, gaggcagcagtagggaatcttc; reverse *Lactobacillus*, ggccagttactacctctatccttcttc; BHQ-1-lacto, atggagcaacgccgc; forward *Bacteroides-Prevotella*, gagaggaaggtcccccac; reverse *Bacteroides-Prevotella*, cgctacttggctggttcag; and VIC-ccattgaccaatattcctcactgctgcct-TAMRA.

**Isolated adipocytes from mesenteric adipose tissue (MAT)**

MATs were weighed, immersed in the digestion medium described below, and cut in small pieces with scissors. Samples were incubated, at 37°C in a shaking bath for 60 min, with 2.5 volumes of Krebs-Henseleit buffer pH 7.4, containing 5 mM glucose, 0.1 μM adenosine (Sigma-Aldrich, St Louis, MO, USA), and 10 g/L lipid-free bovine serum albumin (Merck-Millipore, Billerica, MA USA). This was complemented with 3.5 mkat/L collagenase (LS004196, type I; Worthington Biomedical, Lakewood, NJ, USA). The collagenase-containing digestion buffer was prepared in the cold room (4 .C), and was used within 1 h.

At the end of the digestion process (carried at 37°C), the suspensions were gently sieved using a double layer of nylon mesh hose (plain commercial sheer tight stocking; 90% polyamide, 10% elastomer, parallel woven with 15 den cylindrical single-filament threads; with approximate mean flexible pores in the range of 300 μm), which retained vessel fragments and (eventually) undigested tissue pieces. The smooth crude suspension of isolated cells was left standing for 5 min in stoppered polypropylene syringes (#SS+10ES1, Terumo, Tokyo, Japan), held vertically, at room temperature (22–24°C). The adipocytes floated to form a defined upper layer. Then, the lower aqueous fraction was slowly drained off, capping again the syringe to retain the adipocytes. The cells were washed this way three times, using 2.5 volumes of the buffer each time. Before re-suspending the cells in it, the buffer was subjected to 30 s vortexing, to allow for equilibration with air oxygen. The final supernatant fraction contained intact adipocytes and a thin layer of free fat from broken cells. After the final washing, 400 μL aliquots of the cells’ fraction were taken for incubation. The samples were slowly extracted from the central part of the adipocytes’ layer, trying not to disturb the thin-floating lipid layer. The adipocytes were manipulated and maintained at room temperature for a time as short as possible, and used immediately after the final washing.

**NASH-V-CM/TNFα-simulated cytokines release from rat MAT-derived adipocytes**

The effects of buffer or TNFα (10, 25, 50 ng/mL) pre-treatments for the cytokine [IL-6 and MCP-1] release in supernatant of lean-V rat adipocytes (5×10^5^) at 0h, 2h, 4h and 6h were measured. Preliminary study revealed that the TNFα-stimulated cytokines release was maximized at 4h after the stimulation with 25ng/mL of TNFα. So, this dose (25ng/mL) and duration of TNFα was used to assess the effects of 1,25(OH)_2_D_3_ (vit. D, 10^-9^M and 10^-7^M) on TNFα-stimulated cytokines releases from lean-V rat adipocytes. The similar evaluation for NASH-V-CM effects were followed the same protocols as TNFα group.

***In vitro* effects of calcitriol on NASH-V-CM/TNFα-induced caco-2 cell monolayer barrier dysfunction**

Caco-2 cells between passages 20-45 were cultured in high glucose DMEM supplemented with 10% (v/v) FBS, 1% (v/v) MEM NEAA, and 1% (v/v) penicillin/streptomycin. Cells were incubated at 37℃ and 5% CO_2_, and were sub-cultured at 80-90% confluence every 3-4 days. Near confluence, cells were detached with trypsin, counted, and seeded at a density of 2×10^5^ cells/mL onto polycarbonate membrane Traswell inserts with 0.4μm pore size (Corning, Inc; Lowell, MA). Cells were cultured for 21 days to reach differentiation, and growth media was refreshed every 2-3 days. In differentiated Caco-2 monolayers, buffer, NASH-V-CM, NASH-V-CM+1,25(OH)_2_D_3_ (vit.D, 10^-9^M, 2 h prior to NASH-V-CM) and NASH-V-CM+vit.D (10^-7^M), TNFα (25ng/mL), TNFα+vit.D (10^-9^M) and TNFα+vit.D (10^-7^M) were applied to the apical and basolateral compartments for 48 h.

**References**

1. [Jiang T](https://www.ncbi.nlm.nih.gov/pubmed/?term=Jiang%20T%5BAuthor%5D&cauthor=true&cauthor_uid=26938554), [Gao X](https://www.ncbi.nlm.nih.gov/pubmed/?term=Gao%20X%5BAuthor%5D&cauthor=true&cauthor_uid=26938554), [Wu C](https://www.ncbi.nlm.nih.gov/pubmed/?term=Wu%20C%5BAuthor%5D&cauthor=true&cauthor_uid=26938554), [Tian F](https://www.ncbi.nlm.nih.gov/pubmed/?term=Tian%20F%5BAuthor%5D&cauthor=true&cauthor_uid=26938554), [Lei Q](https://www.ncbi.nlm.nih.gov/pubmed/?term=Lei%20Q%5BAuthor%5D&cauthor=true&cauthor_uid=26938554), Bi J, et al. Apple-derived pectin modulates gut microbiota, improves gut barrier function, and attenuates metabolic endotoxemia in rats with diet-induced obesity. [Nutrients](https://www.ncbi.nlm.nih.gov/pubmed/26938554). 2016:8(3);126-145. doi: 10.3390/nu8030126.
2. [Wang JH](https://www.ncbi.nlm.nih.gov/pubmed/?term=Wang%20JH%5BAuthor%5D&cauthor=true&cauthor_uid=24475077), [Bose S](https://www.ncbi.nlm.nih.gov/pubmed/?term=Bose%20S%5BAuthor%5D&cauthor=true&cauthor_uid=24475077), [Kim GC](https://www.ncbi.nlm.nih.gov/pubmed/?term=Kim%20GC%5BAuthor%5D&cauthor=true&cauthor_uid=24475077), [Hong SU](https://www.ncbi.nlm.nih.gov/pubmed/?term=Hong%20SU%5BAuthor%5D&cauthor=true&cauthor_uid=24475077), [Kim JH](https://www.ncbi.nlm.nih.gov/pubmed/?term=Kim%20JH%5BAuthor%5D&cauthor=true&cauthor_uid=24475077), Kim JE, et al. Flos lonicera ameliorates obesity and associated endotoxemia in rats through modulation of gut permeability and intestinal microbiota. [PLoS One](https://www.ncbi.nlm.nih.gov/pubmed/24475077). 2014:9(1);e86117-e86130. doi: 10.1371/journal.pone.0086117.

# [Gäbele E](https://www.ncbi.nlm.nih.gov/pubmed/?term=G%C3%A4bele%20E%5BAuthor%5D&cauthor=true&cauthor_uid=21703208), [Dostert K](https://www.ncbi.nlm.nih.gov/pubmed/?term=Dostert%20K%5BAuthor%5D&cauthor=true&cauthor_uid=21703208), [Hofmann C](https://www.ncbi.nlm.nih.gov/pubmed/?term=Hofmann%20C%5BAuthor%5D&cauthor=true&cauthor_uid=21703208), [Wiest R](https://www.ncbi.nlm.nih.gov/pubmed/?term=Wiest%20R%5BAuthor%5D&cauthor=true&cauthor_uid=21703208), [Schölmerich J](https://www.ncbi.nlm.nih.gov/pubmed/?term=Sch%C3%B6lmerich%20J%5BAuthor%5D&cauthor=true&cauthor_uid=21703208), [Hellerbrand C](https://www.ncbi.nlm.nih.gov/pubmed/?term=Hellerbrand%20C%5BAuthor%5D&cauthor=true&cauthor_uid=21703208), et al. DSS induced colitis increases portal LPS levels and enhances hepatic inflammation and fibrogenesis in experimental NASH. J Hepatol 2011:55(6);1391-1399. doi: 10.1016/j.jhep.2011.02.035.
