# Supplementary material for: Chronic calcitriol supplementation improves the inflammatory profiles of circulating monocytes and the associated intestinal/adipose tissue alteration in a diet-induced steatohepatitis rat model
Source: PLoS One. 2018 Apr 23;13(4):e0194867. doi: 10.1371/journal.pone.0194867 (PMC5912737; doi:10.1371/journal.pone.0194867)
Supplement: S2 File — (DOCX) [file pone.0194867.s004.docx]

**A protocol for isolation of adipocytes from rat mesenteric adipose tissue (MAT)**

**Yen-Bo Su, Tzu-Hao Li, Ying-Ying Yang**

**Abstract**

Isolation of adipocytes from adipose tissue is a well-established technique for evaluation of the direct *in vitro* effects of various circulating factors on adipocytes in animal adipose tissue, which was performed by measurement of the changes in release of mediators and levels of protein/*mRNA* after acute incubation of specific pathogenic agents. Meanwhile, the interaction between adipocytes and specific cells in animals can be achieved by using supernatant of specific cultured cells as condition medium for cultured MAT-derived adipocytes. Through these *in vitro* approachs, abovementioned *in vivo* mechanisms can be validated. In this study, we provided a step-by-step procedure of isolation of adipocytes from MAT.

**Citation:** Yen-Bo Su, Tzu-Hao Li, Ying-Ying Yang A protocol for isolation of adipocytes from rat mesenteric adipose tissue (MAT) protocols.io

dx.doi.org/10.17504/protocols.io.nashd8

**Protocols**

| 1. Under anaesthetic (pentobarbital, 45 mg/kg), rats were subjected to pre-determined experiments. |
| --- |
| 1. At the end of all *in vivo* experiments, the required rat tissue samples and mesenteric adipose tissue (MAT, the fat surrounding the gastrointestinal tract from the gastroesophageal sphincter to the end of the rectum) were aseptically dissected and collected. |
| 1. MATs were weighed, immersed in the digestion medium described below, and cut in small pieces with scissors. Samples were incubated, at 37°C in a shaking bath for 60 min, with 2.5 volumes of Krebs-Henseleit buffer pH 7.4, containing 5 mM glucose, 0.1 μM adenosine (Sigma-Aldrich, St Louis, MO, USA), and 10 g/L lipid-free bovine serum albumin (Merck-Millipore, Billerica, MA USA). This was complemented with 3.5 mkat/L collagenase (LS004196, type I; Worthington Biomedical, Lakewood, NJ, USA). The collagenase-containing digestion buffer was prepared in the cold room (4 .C), and was used within 1 h. |
| 1. At the end of the digestion process (carried at 37°C), the suspensions were gently sieved using a double layer of nylon mesh hose (plain commercial sheer tight stocking; 90% polyamide, 10% elastomer, parallel woven with 15 den cylindrical single-filament threads; with approximate mean flexible pores in the range of 300 μm), which retained vessel fragments and (eventually) undigested tissue pieces. |
| 1. The smooth crude suspension of isolated cells was left standing for 5 min in stoppered polypropylene syringes (#SS+10ES1, Terumo, Tokyo, Japan), held vertically, at room temperature (22–24°C). The adipocytes floated to form a defined upper layer. Then, the lower aqueous fraction was slowly drained off, capping again the syringe to retain the adipocytes. |
| 1. The cells were washed this way three times, using 2.5 volumes of the buffer each time. Before re-suspending the cells in it, the buffer was subjected to 30s vortexing, to allow for equilibration with air oxygen. The final supernatant fraction contained intact adipocytes and a thin layer of free fat from broken cells. |
| 1. After the final washing, 400 μL aliquots of the cells’ fraction were taken for incubation. The samples were slowly extracted from the central part of the adipocytes’ layer, trying not to disturb the thin-floating lipid layer. The adipocytes were manipulated and maintained at room temperature for a time as short as possible, and used immediately after the final washing. |
